# General Practitioner Hubs and Community Mental Health Team Clinics in Primary Care; Moving Towards Integrated Neighbourhood Care – What Do GPs Want?

**DOI:** 10.1192/bjo.2026.11482

**Published:** 2026-06-30

**Authors:** Adarsh Shetty, Andreena Aloysius

**Affiliations:** Cornwall Partnership NHS Foundation Trust, Liskeard, United Kingdom

## Abstract

**Aims:**

The aim of this quality improvement project was to optimise existing General Practitioner (GP) mental health hub meeting structures for better efficiency, implementing new hub meetings in non-participating surgeries within the primary care network and establishing a framework for satellite psychiatry clinics in GP surgeries.

**Methods:**

The target population consisted of general practitioners in 6 GP surgeries within the North and East Cornwall Primary Care Network (PCN). Between October to November 2024, a structured 12-point electronic survey was distributed to 34 general practitioners to capture both quantitative trends and qualitative feedback. The survey focused on five domains of Community Mental Health Team (CMHT) input into primary care including feasibility, demand, barriers to engagement, logistical preferences (face to face vs virtual) and clinical scope. A reminder email was sent after 4 weeks.

**Results:**

A response rate of 35% was achieved. Out of 6 GP surgeries, 5 had pre-existing GP hub meetings. Two GPs (out of a total of 12 respondents) reported that they did not need a GPHub meeting. 33% of respondents preferred a combination of virtual and face to face meetings, 25% preferred these meetings to be held at the GP surgery and 17% preferred alternating between the GP surgery and CMHT base. Of GP surgeries that had existing hub meetings, 58% preferred the meetings to be monthly for 1 hour each time. Common mental health issues encountered by general practitioners in their settings were mood disorders (24%), anxiety disorder (22%), substance and alcohol misuse (20%), trauma-related experiences/post-traumatic stress disorder PTSD (17%), and psychosis (2%). When offered the option, 67% expressed a wish to have a satellite clinic with a psychiatrist at the GP surgery.

**Conclusion:**

This QI project successfully standardized and expanded the collaboration between the CMHT and GPs in this patch of East Cornwall. By aligning meeting frequency and content with GP preferences, all 6 GP surgeries now participate in either monthly or 3-monthly GP hub meetings. Face to face GP hub meetings are now established in all but one of the surgeries. The demand for closer integration has resulted in the successful establishment of psychiatric satellite clinics in two surgeries, with plans to establish these in all six surgeries.

